# Assessment of U.S. Health Care Utilization Patterns Among Recently Resettled Refugees Using Data from the 2016 Annual Survey of Refugees

**DOI:** 10.1089/heq.2020.0099

**Published:** 2021-05-13

**Authors:** Emma E. Seagle, Curi Kim, Emily S. Jentes

**Affiliations:** ^1^ Division of Global Migration and Quarantine, Centers for Disease Control and Prevention, Atlanta, Georgia, USA.; ^2^ CDC/CSTE Applied Epidemiology Fellowship Program, Atlanta, Georgia, USA.; ^3^ Office of Refugee Resettlement, Administration for Children and Families, Department of Health and Human Services, Washington, District of Columbia, USA.

**Keywords:** refugee, health care utilization, health, resettlement

## Abstract

**Purpose:** Little is known regarding the health care utilization patterns of refugees resettled in the United States. We analyzed the Annual Survey of Refugees (ASR), a nationally representative survey of recently resettled refugees, to assess these patterns.

**Methods:** Anonymized 2016 ASR data were examined for refugees 16 years old who arrived from 2011 to 2014.

**Results:** Refugees most often used private physicians (34%), health clinics (19%), and emergency rooms (14%). Approximately 15% reported no regular source of care, and 34% had health insurance for 1 month of the prior year.

**Conclusion:** Results indicate differing health care use and coverage, revealing opportunities for educational interventions.

## Introduction

Tens of thousands of refugees resettle in the United States annually through the United States Refugee Admissions Program (USRAP).^1^ While the domestic medical screening (an examination recommended by the U.S. Centers for Disease Control and Prevention) should be performed soon after arrival and link refugees to primary care providers for ongoing health care, health disparities persist >1 year postarrival across refugee populations and between refugees and nonrefugees.^25^ Understanding health care utilization patterns post-resettlement is key to developing interventions aimed at reducing disparities and improving health equity.

Prior analyses have examined how resettled refugees seek care (noting limited long-term follow-up), barriers to accessing services (including language and the complexity of U.S. systems), and insurance coverage disparities (some estimates exceeding 40% uninsured).^46^ Yet, these analyses have been limited in geographic scope and populations included. Updating these analyses is also critical given the ever-evolving refugee context.

The Annual Survey of Refugees (ASR) helps fill this knowledge gap. The ASR is a U.S. government-sponsored, nationally representative survey of recently resettled USRAP refugees.^7^ Managed by the Office of Refugee Resettlement (ORR; Administration for Children and Families, Department of Health and Human Services) since the 1980s, the ASR collects information on English language learning, workforce participation, and progress toward establishing permanent residence. Although generally not health-focused, the survey collects some health status and health care utilization data.^7^ Historically, ASR survey data sets have not been publicly available. However, the 2016 ASR data set was anonymized and made available publicly in 2018. We examined health care utilization data patterns among USRAP refugees 15 years post-resettlement.

## Methods

### Data collection

We conducted a secondary analysis using data collected from the 2016 ASR. Conducted in early 2017, the 2016 ASR used a cross-sectional design using stratified probability sampling of refugees arriving from 2011 through 2015 fiscal years (FY; U.S. federal FY: October through September).^7^ During this period, 324,508 refugees resettled in the United States, representing 138 countries and >200 languages.^7^ The telephone survey was interviewer-administered in 17 languages, resulting in a population coverage of 77%. ORR's Refugee Arrivals Data System served as the sampling frame, and the principal applicant (family member whose refugee case is the basis for admission) as the sampling unit.^7^ The principal applicant answered as a proxy for other household members (response rate: 24%). An introductory letter was mailed to respondents; 10 attempts were made to reach respondents.^7^

### Participants

Refugees 16 years old arriving from FYs 2011 through 2014 were included. At the time the survey was conducted, some FY 2015 arrivals could have still been receiving Refugee Medical Assistance (RMA; ORR-funded short-term health insurance available to refugees for up to 8 months after arrival), which could have made their responses different from those of FY 20112014 arrivals. Therefore, FY 2015 arrivals were conservatively excluded.

### Measures

The following three survey questions were assessed: usual source of medical care, length of health insurance coverage in the past 12 months, and type of health insurance coverage in the past 12 months (among those who had it). The survey questions, survey response options, and categorization of responses for the current analysis are listed in Table 1.

**Table 1.** Questions and Response Options Included in Analysis of 2016 Annual Survey of Refugees

Topic	Question from survey^a^	Response options from survey^a^	Categorizations for current analysis
Usual source of medical care	What is (INSERT NAME)'s usual source of medical care?	No regular source Private physician Emergency room at a hospital Health clinic Folk healer Other (specify)	No regular source Private physician Emergency room at a hospital Health clinic Folk healer Other
Length of health insurance coverage (past 12 months)	In the past 12 months, was (INSERT NAME) covered either by Refugee Medical Assistance, Medicaid, or private health insurance?	Yescovered in all months Nonumber of months not covered (specify, ranges 211) Not covered1 month or less Not covered in any month	Continuously covered (12 months) Partially covered (211 months) Minimally covered (01 month)
Type of health insurance (past 12 months)	What type of health insurance coverage did (INSERT NAME) have in the past 12 months?	Insurance through own or family member's employment Private insurance unrelated to employment Medicaid or Refugee Medical Assistance^b^ Other government health care Other insurance	Private (insurance via employment/family's employment, other private insurance) Public (Medicaid, Refugee Medical Assistance^b^, other government source) Other

^a^ Source: 2016 Annual Survey of Refugees, 2019.^7^

^b^ Office of Refugee Resettlement-funded short-term health insurance available to all refugees for up to eight months after arrival.

### Analysis

An individual-level analysis using SAS 9.4 (SAS Institute, Cary, NC, USA) was conducted to describe health care utilization patterns using weighted percentages and confidence intervals (95% CI). Replicate weights (calculated by poststratification raking, provided within the data set) were used to account for differential selection probabilities and differences in respondent and nonrespondent characteristics. This assessment was determined by the Centers for Disease Control and Prevention not to involve human subjects and therefore did not require Internal Review Board approvals.

## Results

The 2016 ASR data set included 3225 refugees 16 years old. Upon exclusion of FY 2015 arrivals, the current analysis included 2411 individuals. Approximately 46% (CI: 4547) were female. Median age was 34 years (range: 1675). Common respondent-reported countries of birth included Iraq, Burma/Thailand (combined for analysis), Bhutan/Nepal (also combined), and Somalia, which together comprised >70% of respondents (Table 2; >10 countries were reported). By U.S. census region, 32% resettled in the South (Northeast: 17%, Midwest: 25%, West: 25%).

**Table 2.** Demographic Characteristics of U.S. Refugee Arrivals 16 Years Old (Fiscal Years 20112014^a^)

Demographic characteristic	Weighted percent (95% CI percent)^b^
Age at survey completion	
1624 years old	20.3 (18.821.8)
2539 years old	39.1 (37.041.1)
4054 years old	20.5 (18.822.2)
55+ years old	13.3 (12.114.4)
Unknown/refused	6.9 (5.78.1)
Sex	
Female	45.7 (44.646.9)
Male	54.2 (53.055.4)
Unknown/refused	0.1 (0.00.3)
Arrival year (fiscal year)^a^	
2011	25.8 (23.428.3)
2012	25.1 (22.627.5)
2013	25.8 (23.827.8)
2014	23.3 (21.625.0)
Country of birth	
Bhutan/Nepal^c^	20.8 (20.121.4)
Burma/Thailand^c^	21.9 (20.323.5)
Cuba	6.2 (6.06.3)
Democratic Republic of the Congo	5.2 (4.16.4)
Iran	6.1 (5.26.9)
Iraq	23.3 (21.325.3)
Somalia	7.4 (6.58.3)
Other^d^	9.0 (7.410.6)
Unknown/refused	0.2 (0.00.5)

^a^ United States federal fiscal year: October through September.

^b^ Weights calculated using poststratification raking based on demographic factors (source: 2016 Annual Survey of Refugees, 2019^7^).

^c^ Combined because same ethnicity (very small numbers from Nepal and Thailand).

^d^ Other: categorized in original data set (countries included in this category are unknown to data user).

CI, confidence interval.

### Sources of care

**FIG. 1.**
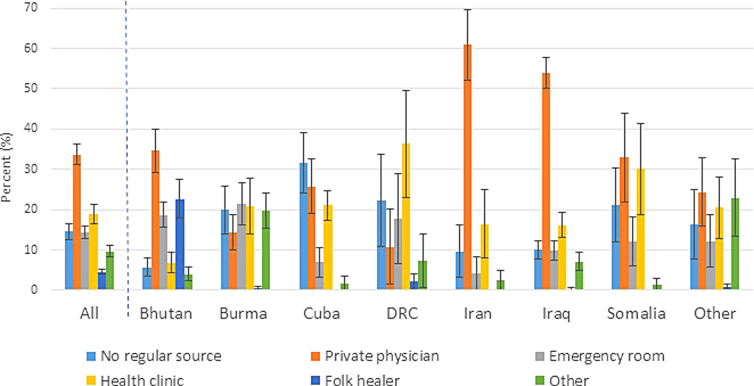
Usual source of health care among U.S. refugee arrivals 16 years old (Fiscal Years 20112014^a^), overall and by country of birth^b^ (weighted percent, 95% confidence interval). ^a^United States federal fiscal year: October through September; ^b^Nepal included with Bhutan; Thailand included with Burma; combined because same ethnicity (very small numbers from Nepal and Thailand); DRC=Democratic Republic of the Congo; other countries categorized as other in original data set (countries included in this category are unknown to data user).

In the United States, refugees most often used private physicians (34%, CI: 3136), health clinics (19%, CI: 1721), and emergency rooms (14%, CI: 1316, Table 3). Approximately 15% (CI: 1317) reported no regular source of care. Individuals from Iran and Iraq reported a meaningfully higher proportion using private physicians, whereas Bhutanese refugees reported a higher usage of folk healers (Fig. 1; folk healer definition unavailable in documentation). Of the 4.6% using folk healers, 92% were Bhutanese. Arrivals from Cuba reported a higher proportion with no regular source of care, particularly those 2554 years old. Overall, males reported that they had no usual source of care more often than females (19%, CI: 1621 vs. 10%, CI: 712). Females were more likely to seek care at health clinics compared to males (23%, CI: 1926 vs. 16%, CI: 1319). By census region, no regular source of care was common in the South; private physician use was common in the West.

**Table 3.** Health Care Utilization Patterns of U.S. Refugee Arrivals 16 Years Old (Fiscal Years 20112014^a^)

Health care utilization	Weighted percent (95% CI percent)^b^
Usual source of medical care	
Private physician	33.7 (31.236.1)
Health clinic	18.9 (16.621.3)
Emergency room at a hospital	14.4 (12.815.9)
Folk healer	4.6 (4.05.7)
Other	9.7 (8.410.9)
No regular source	14.6 (12.616.6)
Unknown/refused	4.1 (2.95.4)
Length of health insurance coverage (past 12 months)	
Continuously covered (12 months)	54.5 (52.456.6)
Partially covered (211 months)	6.0 (5.16.9)
Minimally covered (01 month)^c^	33.9 (31.736.1)
Unknown/refused	5.6 (4.27.0)
Type of health insurance (past 12 months)^d,e^	
Private	16.0 (14.018.0)
Public	62.7 (59.466.0)
Other	10.8 (8.712.9)
>1 type	3.9 (2.95.0)
Unknown/refused	6.6 (5.08.2)

^a^ United States federal fiscal year: October through September.

^b^ Weights calculated using poststratification raking based on demographic factors (source: 2016 Annual Survey of Refugees, 2019^7^).

^c^ Not covered at any time (0 months): 32.4 (30.334.5).

^d^ Private insurance comprised those who responded with insurance via own/family employment or private insurance not from employment; public insurance comprised those who responded with Medicaid, Refugee Medical Assistance (Office of Refugee Resettlement-funded short-term health insurance available to all refugees for up to eight months after arrival), or other government source; other insurance comprised those who responded to question as other insurance.

^e^ Asked only of those who did not answer not covered at any time for length of insurance coverage.

### Length of health insurance coverage

**FIG. 2.**
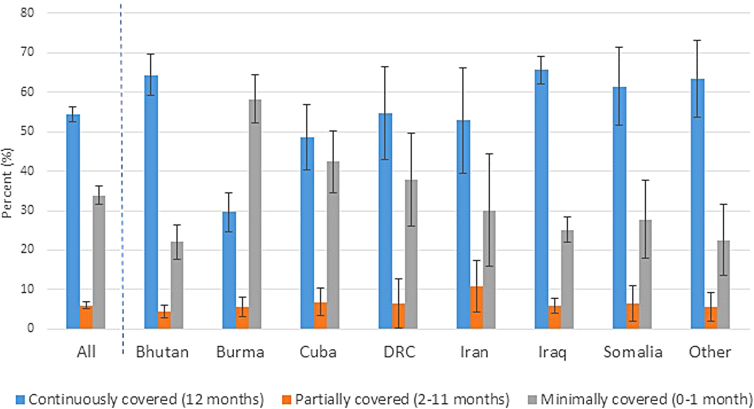
Length of health insurance coverage in 12 months before survey among U.S. refugee arrivals 16 years old (Fiscal Years 20112014^a^), overall and by country of birth^b^ (weighted percent, 95% confidence interval). ^a^United States federal fiscal year: October through September; ^b^Nepal included with Bhutan; Thailand included with Burma; combined because same ethnicity (very small numbers from Nepal and Thailand); DRC=Democratic Republic of the Congo; other countries categorized as other in original data set (countries included in this category are unknown to data user).

Overall, 55% (CI: 5257) were continuously covered with health insurance for the 12 months in the United States before the survey. Nearly 34% (CI: 3236) were covered 1 month (Table 3). Burmese refugees had the lowest proportion with continuous coverage (Fig. 2). Among adults 18 years old, the percentage with health insurance for >1 month generally increased with age. Females tended to have a higher proportion with continuous coverage compared to males (63%, CI: 6066 vs. 48%, CI: 4451). Proportion continually covered also varied by U.S. census region, with lower percentages in the South (continuously covered: 40%, CI: 3645) than in other regions (Northeast: 67%, CI: 6172; Midwest: 58%, CI: 5363; West: 63%, CI: 5868).

### Type of health insurance

**FIG. 3.**
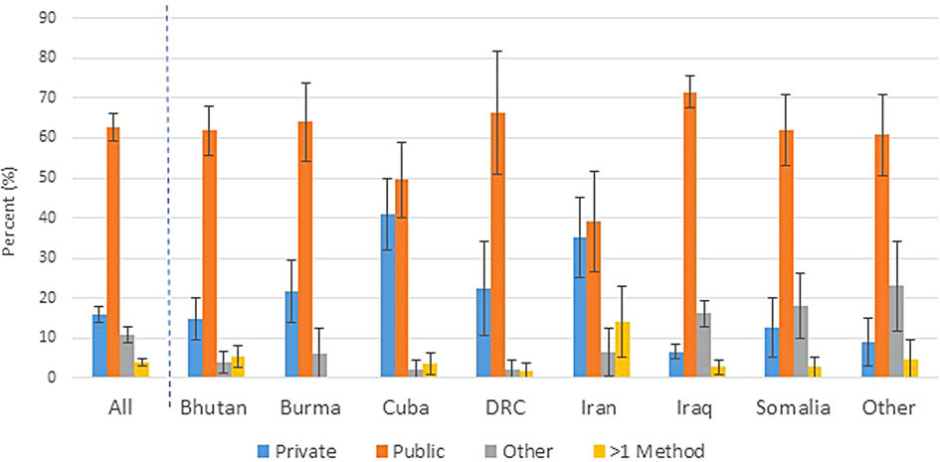
Type of health insurance coverage^a^ among refugee arrivals 16 years old (Fiscal Years 20112014^b^), overall and by country of birth^c^ (weighted percent, 95% confidence interval). ^a^Private insurance comprised those who responded with insurance via own/family employment or private insurance not from employment; public insurance comprised those who responded with Medicaid, Refugee Medical Assistance (Office of Refugee Resettlement-funded short-term health insurance available to all refugees for up to eight months after arrival), or other government source; other insurance comprised those who responded to question as other insurance; excludes those with no health insurance at any time in prior 12 months; ^b^United States federal fiscal year: October through September; ^c^Nepal included with Bhutan; Thailand included with Burma; combined because same ethnicity (very small numbers from Nepal and Thailand); DRC=Democratic Republic of the Congo; other countries categorized as other in original data set (countries included in this category are unknown to data user).

In general, among the 1618 individuals with health insurance in the United States, most respondents (63%, CI: 5966) had public insurance (Table 3). Although overall lower than public insurance, the proportion with private insurance peaked among 2554-year-olds. Females tended to have a higher proportion of public insurance coverage than males (68%, CI: 6373 vs. 57%, CI: 5362). The proportion covered with private health insurance was highest among those from Iran and Cuba (35%, CI: 2545 and 41%, CI: 3250, respectively) (Fig. 3). By census region, private insurance was common in the South; public insurance was common in the Midwest.

## Discussion

Refugees' differing sources of U.S. medical care and health insurance coverage across country of birth, age, and gender reveal disparities in health care access. Most notably, the overall percentage of refugees with no U.S. health insurance coverage was high (32%) compared to the U.S. population (9% uninsured in 2016).^8^ Lower continuous coverage in the Southern U.S. census region was also observed. Medicaid expansion pursuant to the Affordable Care Act (ACA) has been shown to be associated with increased health insurance coverage and health service use.^8,9^ Of the 17 states in the Southern U.S. census region, 11 had not expanded Medicaid eligibility as of January 1, 2016.^10^ Similar patterns of minimal continuous coverage among refugees have been previously noted (49% uninsured in 2003; although ACA-related Medicaid expansion began for many states in January 2014, our analysis indicated that the uninsured rate remained high).^11^ Reasons behind the low coverage remain unclear, but could include difficulty navigating the U.S. health care system, language barriers, or lower health literacy.^12^ Lack of insurance has been associated with an increase in unmet health needs, lack of routine care and preventive services, and worse health outcomes.^13,14^ Further analyses are needed to inform programmatic and educational interventions (such as educational campaigns about how to access affordable health insurance options) aimed at reducing disparities and increasing coverage.

Our analysis indicated that Bhutanese refugees used folk healers in the United States more often than those from other countries. Refugees from Iraq and Iran showed a greater tendency to seek care through a private physician. The latter is potentially related to higher socioeconomic status or education level, affordability (often related to employment status), or familiarity with western medicine.^15^ Regional variations may be associated with differences in location of U.S. resettlement by birth country (e.g., cultural preferences) or availability of resources. Additional investigations are needed to understand how and why certain populations seek care at particular sources. Other analyses have also indicated some refugees' high use of the emergency department, which may indicate lack of regular primary care and access to preventive services resulting in unhealthier outcomes.^16^

Ultimately, understanding where refugees seek care in the United States helps to identify subpopulations in need of targeted education and may inform decision-makers about the efficiency of intervention options aimed at reducing disparities. For instance, health education materials intended for Bhutanese refugees could be distributed to folk healers (those who practice traditional medicine that incorporates cultural aspects) in efforts to reach the target population. No regular source of care could be associated with difficulty navigating the health care system or access barriers (e.g., transportation), pointing to the need for further understanding of these factors to develop effective interventions.

### Limitations

Small sample sizes, particularly after stratification by country of birth, diminished the estimates' precision, and limited the use of modeling to control for confounders (e.g., education, employment/income, and region) and assess gender differences by birth country. Second, those with other as birth country were categorized like this in the original data set, and the countries included in this category are unknown. Because exact arrival dates were unavailable, we could not separate FY 2015 arrivals still eligible for RMA from those no longer eligible. Therefore, we conservatively excluded FY 2015 arrivals. However, this decision likely excluded some eligible for inclusion, thus reducing our sample size and estimate precision. Accuracy of responses likely depended on unknown factors, including the principal applicants' knowledge of their household members' health care utilization and understanding of the U.S. health care system. For the latter, RMA (similar to Medicaid, but available only within 8 months of arrival) is suspected to have been confused with Medicaid, as many respondents >1-year postarrival reported receiving RMA. The potential for question misinterpretation (e.g., RMA vs. Medicaid) or differing understandings/definitions (e.g., folk healer) cannot be ruled out. Given small sample sizes, limited translation, and solely telephone administration, results may not be representative of all refugees. The survey may also not be representative of more recently resettled refugees and comparison to future ASRs is recommended to understand temporal trends.

## Conclusion

Prior analyses of refugees >1 year after U.S. resettlement covered only certain jurisdictional regions or used less representative sampling methodologies, limiting generalizability. This investigation is unique in that it used a nationally representative sample to provide insight into health care utilization patterns >1-year postarrival. Current funding priorities and programmatic interventions tend to focus efforts within the first year of arrival.^11^ Our analysis revealed gaps in health care utilization and coverage >1-year postarrival, which may inform decision-makers seeking to improve disparities in refugee health care utilization. Results can be used to strengthen current systems to increase health care and insurance access, ultimately improving health equity.

## Abbreviations Used

ACA: Affordable Care Act
ASR: Annual Survey of Refugees
CI: confidence intervals
FY: fiscal years
ORR: Office of Refugee Resettlement
RMA: Refugee Medical Assistance
USRAP: United States Refugee Admissions Program
